# Understanding how domestic health policy is integrated into foreign policy in South Africa: a case for accelerating access to antiretroviral medicines

**DOI:** 10.1080/16549716.2017.1339533

**Published:** 2017-07-07

**Authors:** Simon Moeketsi Modisenyane, Stephen James Heinrich Hendricks, Harvey Fineberg

**Affiliations:** ^a^ Faculty of Health Sciences, School of Health Systems and Public Health, University of Pretoria, Pretoria, South Africa; ^b^ President of Gordon and Betty Moore Foundation, Palo Alto, CA, USA

**Keywords:** Global health, global health diplomacy, policy analysis, South Africa, HIV/AIDS

## Abstract

**Background**: South Africa, as an emerging middle-income country, is becoming increasingly influential in global health diplomacy (GHD). However, little empirical research has been conducted to inform arguments for the integration of domestic health into foreign policy by state and non-state actors. This study seeks to address this knowledge gap. It takes the form of an empirical case study which analyses how South Africa integrates domestic health into its foreign policy, using the lens of access to antiretroviral (ARV) medicines.

**Objective**: To explore state and non-state actors’ perceptions regarding how domestic health policy is integrated into foreign policy. The ultimate goal of this study was to achieve better insights into the health and foreign policy processes at the national level.

**Methods**: Employing qualitative approaches, we examined changes in the South African and global AIDS policy environment. Purposive sampling was used to select key informants, a sample of state and non-state actors who participated in in-depth interviews. Secondary data were collected through a systematic literature review of documents retrieved from five electronic databases, including review of key policy documents. Qualitative data were analysed for content. This content was coded, and the codes were collated into tentative categories and sub-categories using Atlas.ti v.7 software.

**Results**: The findings of this work illustrate the interplay among social, political, economic and institutional conditions in determining the success of this integration process. Our study shows that a series of national and external developments, stakeholders, and advocacy efforts and collaboration created these integrative processes. South Africa’s domestic HIV/AIDS constituencies, in partnership with the global advocacy movement, catalysed the mobilization of support for universal access to ARV treatment nationally and globally, and the promotion of access to healthcare as a human right.

**Conclusions**: Transnational networks may influence government’s decision making by providing information and moving issues up the agenda.

## Background

Health policy has changed in the twenty-first century as a result of globalization and the higher priority of health in international political agendas [1–3]. Recognizing the close, interdependent relationship between foreign policy and global health, the foreign ministers of seven countries issued the Oslo Ministerial Declaration, entitled ‘Global health – a pressing foreign policy issue of our time’ [4]. Subsequently, the United Nations General Assembly (UNGA) adopted several resolutions on health and foreign policy. Furthermore, a number of countries, such as the USA, the UK and Norway, launched their national global health strategies to explore how foreign policy could contribute towards tackling important health issues and how a health dimension could benefit foreign policy. Within this context, the term global health diplomacy (GHD) has emerged to describe the political processes that states and non-states use to negotiate about health-related foreign policy issues [5]. Furthermore, looking back over the past 15 years, there has been an increase in the number of actors, processes and initiatives that acknowledge and reflect both the diversity of global power and the need to act together [6]. The north–south divide has changed through undertaking joint but differential responsibilities. In addition, innovative development finance is gaining ground over development aid and there is a renewed commitment to common goals, as reflected in the Sustainable Development Goals (SDGs). The creation of new transnational collaborations between India, Brazil and South Africa (IBSA), and between Brazil, Russia, India, China and South Africa (BRICS), has led to power shifts in political and economic decision making and is transforming international health policies [7].

Therefore, from a public health perspective, it is important to understand how the proliferation of actors, processes, new institutions and initiatives will shape global health governance, its approaches and its values [8]. The main challenge for governments and non-state organizations is in developing a multisectoral and coherent approach to overcome fragmented policy coherence [9].

In light of these challenges, several commentators have tried to introduce a structure to analyse the global health policymaking process. However, many scholars have argued that no single framework offers a fully comprehensive description or understanding of the policymaking process, as each framework answers somewhat different questions. The lack of agreed-upon theoretical and analytical frameworks for GHD means that the current GHD literature is fairly fragmented, and not clearly structured around key research problems or questions [10]. In this article, we use Kingdon’s Multiple Streams Model to explain how a national public health issue is integrated into foreign policy agendas, in particular, access to antiretroviral (ARV) medicines in South Africa [11,12]. While the Kingdon Model contains useful elements, it does not include enough detail on the problem/agenda-setting process, nor does it monitor the quality of policy implementation or measure policy impact. As such, in this article, we use the adapted Walt and Gilson’s (1994) policy analysis triangle as a heuristic device to gather and organize a comprehensive and relevant set of data in five areas [13,14]. A fifth important category has been added by Gagnon and Labonté (2013) to capture data that reveal the potential and actual effects of a policy [13]. These frameworks are not exclusive or theoretically or analytically distinct, but are used together as a useful and novel mechanism by which to analyse and interpret the research findings.

In this article, we focus on the domestic public health policy on access to ARV medicines in South Africa. Access to ARVs was chosen pragmatically as South Africa currently has the largest number of people living with human immunodeficiency virus (HIV) globally. An estimated 6.8 million people are living with HIV, of whom around 3.1 million are currently receiving antiretroviral treatment (ART) [15]. Therefore, this article looks at how a policymaking process is undertaken to integrate domestic public health policy into foreign policy in South Africa, using the lens of access to ARV medicines. The ultimate goal of this study is to achieve better insights into the health and foreign policy processes at the national level.

## Methods

We used a case-study design that included a systematic literature review (of both peer-reviewed and grey literature), analysis of official policy documents and in-depth interviews with key GHD actors. We first conducted a systematic review of literature for South Africa between May 2014 and December 2015. We expanded our search to include literature between 2000 and 2015 because GHD is a relatively new phenomenon which saw the launch of the Oslo Declaration as a commitment to advancing health and well-being on a global scale. We limited our search to studies written in English.

**Figure 1.**
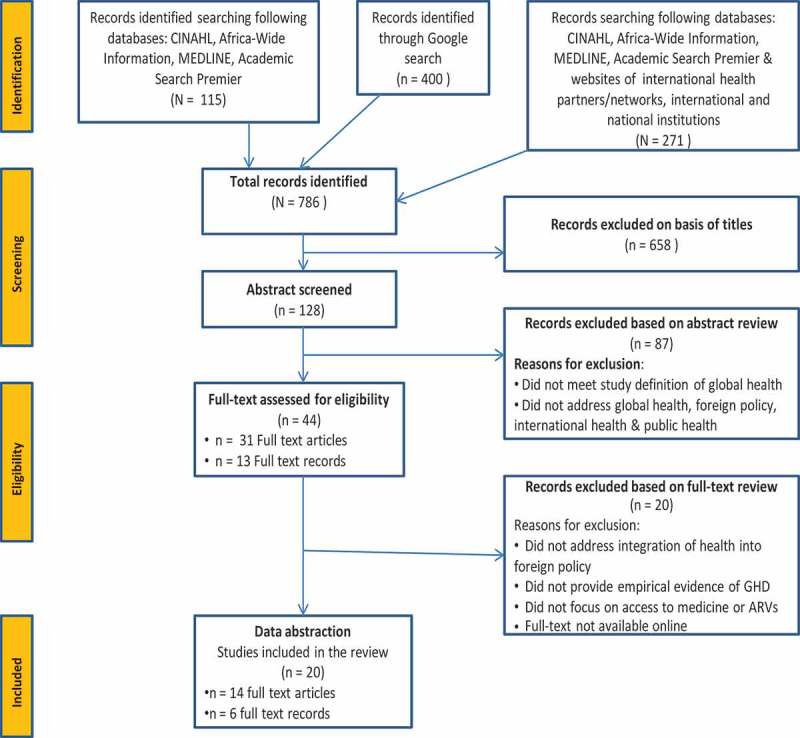
Flow diagram of study selection for the systematic review of published research on global health diplomacy (GHD) in South Africa. ARVs = antiretrovirals.

We used search engines such as Academic Africa-Wide Information, Academic Search Premier, CINAHL and MEDLINE to find literature. We used the key words ‘global health’, ‘diplomacy’, ‘foreign affairs’, ‘global health diplomacy,’ ‘medicines’ and ‘antiretroviral medicines’ (Figure 1). In this article, we focused on South Africa’s GHD policy for access to ARV medicines. As a further check, we conducted six searches using Google Scholar on title and/or abstract, with screening stopping on the tenth page/article which signifies the tenth redundant article in a row.

We manually searched the following key journals in the past year: Health Policy & Planning, World Health Organization Bulletin, The Lancet, Globalization and Health, Health Policy and Global Health Governance. Reference lists of relevant articles, related systematic reviews and key author searches were conducted. Articles were excluded if they had a domestic analysis, reported global epidemiological data, did not focus on health or were summary reports. A final collection of 20 peer-reviewed articles and 25 grey literature articles constituted the data set on which the analysis of this article is based.

Official policy documents relevant to primary cases, South Africa, were reviewed and their content categorized systematically using Walt and Gilson’s heuristic categories [14]. Documents were analysed in terms of their content value and their historical value. All the documents were screened for relevant content. A total of 26 key documents (of which 14 were published policy documents) were used for analysis.

Both SMM and SJHH conducted an independent full-text review of all the articles and policy documents using pre-existing data extraction forms. Disagreements that occurred during the application of the exclusion criteria or data extraction were negotiated until consensus was reached.

We used purposeful sampling [16] to identify and recruit interviewees based on relevant peer-reviewed or grey literature. Interviewees had to have extensive professional networks in global health and development. Additional interviewees were enrolled using snowball sampling until no new concepts emerged. Respondents came from state, non-state and intergovernmental organizations. We conducted 21 interviews, drawn from state and non-state actors with extensive experience (i.e. more than 10 years’ worth) and skills related to GHD, and those involved in the international negotiations for improved access to ARV medicines, as outlined in Table 1. Interviews were conducted between November 2015 and April 2016. All 13 South African interviewees were personally interviewed at convenient times by the lead researcher. Eight international interviewees were interviewed by telephone, via Skype and during the margins of regional and global health events, including the United Nations (UN) High Level Panel on access to medicines in Johannesburg, South Africa, March 2016, and the East, Central and Southern African-Health Community, held in Nairobi, Kenya, April 2016. The interviews lasted for about 45 minutes to 1 hour. Interviewees were recruited using the recruitment letter. Informed consent was obtained before the interviews began. All interviews were tape-recorded and transcribed verbatim.

**Table 1.** Characteristics of key informants from all cases (*n* = 21).

Characteristics		
Affiliation	Government	9 (42)
	NGO	1 (5)
	Academia	3 (14)
	Research	1 (5)
	Think tank	2 (10)
	Private sector	2 (10)
	UN agency	3 (14)
Gender	Male	12 (57)
	Female	9 (43)
Rank	Director and upwards	9 (42)
	CEO	2 (10)
	Scientist or researcher	2 (10)
	Lecturer and upwards	3 (14)
	Policy advisor or analyst	3 (14)
	Diplomat	2 (10)
Cases	Primary	13 (62)
	Background	8 (38)

Data are shown as *n* (%).

NGO = non-governmental organization; UN = United Nations; CEO = chief executive officer.

Data collection and analysis were done concurrently, resulting in continuous interpretations and preliminary reports. Data were analysed for content, coded, and subsequently collated into tentative sub-categories and categories using software Atlas.ti Scientific Software, v.7 [16,17]. Data analysis was continuously discussed by SMM and SJHH to ensure constant comparison of the sub-categories and categories with the original text. To preserve anonymity, names and affiliations are not released. Ethical approval was obtained from the University of Pretoria’s Ethics Committee.

## Results

### Context: health is a human right

In South Africa, the concept of health as a human right is important in both national and international policy [18–21]. The inclusion of health in the South African constitution, which seeks to guarantee individual rights, was supported by former President Mandela, who argued that ‘issues of human rights are central to international relations and an understanding that they extend beyond the political, embracing the economic, social and environmental’ [22].

In 2007, South Africa participated in the formation of the Global Health and Foreign Policy Initiative, where the Oslo Ministerial Declaration identified ‘global health’ as ‘a pressing foreign policy issue of our time’ [4]. The Oslo Declaration refers to human rights several times, accepting that ‘health is a fundamental right of every human being’ and, in line with legal scholarship, that ‘life is the most fundamental of human rights, and that life and health are the most precious assets’ [4]. South Africa has also harnessed its moral power, its own struggle for democracy, its commitment to the promotion of human rights, and its multilateral focus to leverage its own sovereignty and that of weaker states, especially with respect to access to medicines and migration of health professionals [18,19,23,24]. In particular, the South African government has championed for increased access to ARV drugs to provide universal treatment to all HIV-positive people [25]. The role of human rights was emphasized by a non-state actor:

When we were fighting the pharmaceutical (industry) in 1998, we used the human rights argument strongly … (because) … we knew as a new democracy emerging out of apartheid … (with) the strong moral presence of President Mandela … nobody would want to be seen to be resisting the intent of this new government to make medicines affordable to the people who have been marginalized.

Despite strong views on human rights, the politics of solidarity and sovereignty has interfered with international policy. South Africa, like many other African countries, places importance on autonomy and independence, which may overshadow the importance of human rights in the policymaking process [26]. Protecting national sovereignty has led to important transnational issues, such as sex worker health and rights, falling by the wayside. A state actor gave the following example:

We have been pretty silent to HIV related to human rights failures in Africa on sex worker[s], you know even though we do well as a country, it doesn’t seem to be something we need to export.

### Content: health in all policies

South Africa uses a Health in All Policies (HiAP) approach to incorporate health outcomes into non-health-sector policies [7,27,28]. This represents an inter-sectoral approach to developing national and sub-national public policy such that health outcomes are given full consideration by the non-health sectors. South Africa’s National Department of Health’s Strategic Plan aims to ‘prevent disease and reduce its burden, and promote health through a multi stakeholder National Health Commission’ [29]. South Africa’s health-related foreign policy interests are reflected in the Oslo Ministerial Declaration [4]. Achieving these aims will require collaboration and participation in a system of global health governance to ‘influence position and strengthening policy coherence and coordination’. A close examination of the contents of the action agenda highlights the following values:

… [there is an] acknowledgement that globalisation requires new forms of governance in order to ensure sustainable development, social and economic equity, justice, peace, and security. It recognises the need for cooperation and collaboration, a respect for national sovereignty, a sense of shared responsibility, and the attributes of transparency, trust, accountability, and fairness. [4]

In order to promote HiAP, South Africa’s public health experts have participated in various national and global initiatives that have encouraged policymakers to prioritize the health and human rights of disadvantaged people in their policies. This represents a bid to effectively reorientate global health systems towards the goal of healthcare equity [21,30–32]. In South Africa, the negotiation of lower ARV prices and reforms of the pharmaceutical supply chain have resulted in the reduction of ARV prices on the global scale. Thus, South Africa has successfully leveraged its model fight against HIV/acquired immune deficiency syndrome (AIDS) and tuberculosis (TB) to promote South–South collaboration and leadership. For example, a rapid scaling-up of ART services in South Africa resulted in a four-fold increase in the number of people receiving ART services between 2004 and 2011, as women, men and children gained greater access to ART [33]. In addition, the number of TB deaths in South Africa declined from 70,000 in 2009 to fewer than 40,000 in 2014 [34,35]. This, in turn, has enhanced the country’s reputation and influence at the global level, and granted South Africa a more significant voice in global health governance systems [36–38].

### Agenda-setting phase: shaping and determining the issues of importance

South Africa has participated in various global initiatives to raise the profile of health issues of importance in general, and access to medicines in particular, within the world of foreign policy development [39–42]. Nationally, South Africa successfully drew the attention of the world when it passed a law allowing cheap generic drugs to be imported to promote universal access to medicines. In 2001, South Africa successfully put access to medicine in the global spotlight through the Pharmaceutical Manufacturers Association (PMA) case in which 39 pharmaceutical companies took the South African government to court regarding the government’s intention to reform its Medicines Act [38]. In this case, the South African government, backed by various civil society organizations (CSOs) and transnational networks, was instrumental in highlighting access to medicines as a global policy issue [38,42]. The case was followed by various high-level meetings and processes which kept attention on the issue and motivated the development and adoption of the Doha Declaration in 2001, as well as the health-related flexibilities contained in the Trade-Related Aspects of Intellectual Property Rights (TRIPS) agreement. As explained by one state interviewee:

our access to medicines is centered mostly, mostly to a large extent, not around politics of getting medicines, maybe a little around infrastructure for absorption of medicines … but largely access to medicines for developing countries including ourselves is the cost … remember in medicine one of the biggest factor[s] on the price of medicine is what they call the ability to pay … ability to pay in south Africa is quite high.

However, the reason why South Africa went for years without the option of sourcing generics was because some pharmaceutical companies have managed to limit generic competition by entering into voluntary licensing agreements with the majority of Indian generic manufacturers, and these manufacturers were unable to export generic medicines to middle-income countries such as South Africa and Brazil [43]. Furthermore, although Gilead did not patent tenofovir disoproxil fumarate (TDF) in South Africa, it awarded the licensing agreement to Aspen Pharmacare and the overall result was that TDF remained a monopoly (single-source) product [43,44]. In addition, while experts involved in ARV price negotiations and forecasting anticipated reduction in the price of TDF, three major obstacles to South Africa’s inability to access lower prices were the ARV tendering process for 2008, legal restrictions and medicine registration [43,44]. For instance, the first ARV tender, concluded in 2004, locked the South African government into a 3 year agreement with manufacturers. In addition, certain bidding clauses, such as the need for bidders to submit a copy of the actual patent or an agreement with the patent holder, were potentially highly restrictive. Regarding medicine registration, despite affordable, quality-assured, generic versions of TDF becoming available in 2006, the medicine took several more years to be registered because of the complexity of two pharmaceutical companies owning different patents [43,44].

Globally, South Africa, as part of the Oslo Group, drove the development of the first three resolutions for the integration of global health into foreign policy at the UNGA; namely, Resolutions A/63/33, 2008; A/64/108, 2009 and A/65/95, 2010 [45]. These resolutions afford more attention to global health in the development of a number of nations’ foreign policies. Several countries have already launched national global health strategies – such as Switzerland (2006), the UK (2008), Norway and Japan (2010) and Sweden (2011), as well as the European Union. These resolutions were followed by various high-level meetings and processes that kept the world’s attention on these issues and led to the adoption of various further resolutions and reports by the United Nations Secretary General (UNSG) on the integration of global health into foreign policy at the UNGA itself.

### Policy development process: multilevel and multisectoral process

In South Africa, the process of integrating health into foreign policy within a multisectoral structure was directed by two main principles: ‘inclusiveness’ and ‘human-rights-centredness’ [25,30,32,46]. For example, during the development of various aspects of South Africa’s National Strategic Plan for HIV/AIDS (the NSP), the broad consultation and consensus-building process ensured that various important stakeholders were included as actors in the policymaking phase [46]. The literature highlights the importance of mobilizing various stakeholders and community responses in order to facilitate improved access to health and healthcare services in South Africa [27,30,47,48]. Experience has shown the importance of rooting HIV projects at the community level, and then scaling these activities up to include civil society, business and public health initiatives. This bottom–up approach has led to considerable innovation and expansion with regard to HIV-prevention endeavours in South Africa. For example, the Global Fund has seen fit to support one of the largest and most innovative HIV prevention programmes in the world, in Khayelitsha (in South Africa’s Western Cape province) [25,48]. Transferring these local innovations to national programmes and ensuring sustainability remains a challenge to South African initiatives. However, South African initiatives may be hamstrung by the lack of communication between key stakeholders from civil society, the private sector and academia. Furthermore, South African policymakers have been criticized by CSOs for being ‘too accommodating’ of the needs of the developed countries during TRIPS negotiations [49].

### Policy implementation process: politics and power in context

Even though South Africa has participated in various forums for the integration of health into foreign policy, especially to improve access to medicines in South Africa, the country has faced many challenges with regard to the implementation of HIV/AIDS policy [50–52]. At the national level, these challenges are related to the sheer content, as well as to power struggles between different levels of government, the sluggish development of programme infrastructure, the lack of global experience among policy entrepreneurs and a lack of necessary systems. As outlined by one of state actor:

You can develop a policy but there is a also capacity to implement the policy … the other thing is implementation demands that you have systems in place, your systems from regulatory, patent protection, warehousing, those are not easy things.

While the Foreign Policy and Global Health (FPGH) initiative and its Oslo Ministerial Declaration gained prominence by reflecting the rise of health as a foreign policy issue, the ultimate impact of the FPGH appears limited [51,52]. One of the criticisms of the FPGH was that it lacked a formal coordinating structure or permanent secretariat to manage the implementation of the action points on its agenda. The grey literature seems to suggest that the Oslo Group was based on personal links and common interests between the Ministers of Health and Foreign Policy [51]. Now that most of the original ministers who were signatories of the Oslo Declaration (including Dr Dlamini-Zuma) are no longer in power, there has been a distinct decline in South Africa’s investment in the FPGH. Furthermore, there has been a lack of visible implementation of the Oslo Declaration at the national level. Nevertheless, while it is clear that the Oslo Declaration was not the catalyst for the recent rise in global interest in health and foreign policy, it seems undeniable that it inspired the more effective UN Resolution A/64/108 on global health and foreign policy [51].

### Actors: managing diversity of health partnerships in GHD

In South Africa, the integration of health into foreign policy has been driven by a diverse array of health partnerships working in collaboration with the government [25,27,30,35,46,48]. For example, inspired by their new constitution, South African CSOs (and the Treatment Action Campaign in particular) demanded that the South African government introduce a national programme for the prevention of mother-to-child transmission (PMTCT) of HIV [30]. The government argued that it could not implement PMTCT due to the price of drugs such as azidothymidine (AZT). However, the Treatment Action Campaign (TAC) used the South African constitution to argue that people’s right to health should take precedence over the profit margin of international pharmaceutical companies. This case serves as an example of CSOs’ ability to use the law and governing institutions to create space for policy reform. Such cases ultimately resulted in the integration of local public health issues (such as access to ARVs) into foreign policy debates.

The literature reviewed for this study demonstrates the importance of partnerships at the community level in the bid to improve access to ARVs, and to successfully integrate health issues into foreign policy [42,48]. For example, the TAC built on the success of its fluconazole campaign and mobilized local South Africans on the ground and all of its global contacts (in particular its close ally, Doctors Without Borders) to prove to the South African government that treating HIV/AIDS with cheap generic drugs was cost-effective and could even be achieved in areas with limited health facilities in resource-poor settings [48]. Although, ultimately, the South African government would not issue compulsory licences for medicines, the TAC-driven pilot programme in Khayelitsha proved to be a great success. Brazil subsequently offered to assist South Africa, through technology transfer, to establish a South African state-owned pharmaceutical plant. However, the South African government eventually negotiated with global pharmaceutical companies to obtain the technology transfer necessary to produce cheap generics for South Africa through the private sector, such as Aspen Pharmacare.

The literature reviewed for this study shows that the only way to effectively respond to the AIDS epidemic is by involving all sectors of society, including business. The South African business sector recognizes that engaging in the HIV/AIDS response is part of their corporate and social responsibility [15]. Three companies in South Africa – Anglo American, BHP Billiton and Eskom – have been providing their HIV-positive employees with ART. This has shown that partnerships between industry, labour, government, communities and civil society represent the most promising approach to effectively addressing the global AIDS epidemic.

By the same token, the literature also demonstrates how other developing countries such as Brazil, India and Thailand have been successful in influencing HIV/AIDS policy reforms in South Africa as ‘significant others’ in various partnerships [38,42,46,53]. For example, the PMA case of 2001 harnessed strong leadership from the Brazilian government, strong activist mobilization and a number of other factors, and laid the groundwork for the Doha Declaration of 2001. The Declaration clarified much of the legal uncertainty that had existed around the TRIPS agreement, particularly with regard to flexibility in domestic laws designed to safeguard public health and the affordability of drugs. In the Declaration, the World Trade Organization (WTO) member countries state the following:

We affirm that the agreement [TRIPS] can and should be interpreted and implemented in a manner supportive of WTO members’ right to protect public health and, in particular, promote access to medicines for all. [54]

Furthermore, the literature shows that transnational networks have successfully employed the strategy of ‘issue linkage’ to assist South Africa in reforming its policy on HIV/AIDS [30,38,42]. For example, in February 1998, 39 (mostly multinational) pharmaceutical manufacturers filed a suit against the South African government, alleging that the Medicines and Related Substances Control Amendment Act No. 90 of 1997 (known as ‘the Amendment Act’) violated both TRIPS and the South African constitution [30]. By the time the case finally reached the courtroom in May 2000, the drug companies could no longer rely on the support of their home governments.

In addition, the literature demonstrates that even though South Africa is an upper middle-income country, it continues to receive significant funding support from the Global Fund and the US President’s Emergency Plan for AIDS Relief (PEPFAR) owing to its high disease burden. South Africa’s financial contribution to the HIV/AIDS programme has increased from 76% in 2011 to 80% in 2013 [55]. The second highest HIV contribution over the same period was made by the US government, although this contribution decreased from 22% (of the total) in 2011 to 17% in 2013. The Global Fund’s contribution to HIV- and TB-related causes in South Africa rose from 1% of the total in 2011 to 3% in 2013 owing to initial delays in the start-up of the Single Stream Funding grant. Declining donor funding will be detrimental to health development in South Africa since the government amended its HIV treatment threshold to a CD4 cell count of 500 cells/mm^3^ in May 2016 and committed to reaching its 90–90–90 targets of the Joint UN Programme on HIV/AIDS, both of which are bold and ambitious steps that can only be accomplished with significant resource input.

Despite assurances by the US Embassy in Pretoria that there is ‘every expectation that funding levels will continue’, there is growing anticipation that this assistance will decline and ultimately flatline [55]. Although relatively little US funding is used for ARV purchases or service delivery, the vast majority is earmarked for essential training and technical assistance. For example, the US government, through its PEPFAR programme, worked in partnership with South Africa’s national, provincial and local governments, non-governmental and faith-based organizations, academics and the private sector to improve various life-saving HIV/AIDS prevention, care and treatment services across South Africa. Some of the interviewees in this study supported the view that development health assistance is the driving force behind South Africa’s many recent achievements in the area of HIV/AIDS. As one interviewee stated:

… for example to be quite honest if we didn’t have PEPFAR support in South Africa, we wouldn’t have made gains that we have made right … we would have made gains for sure because we have put our own money as well, …

To ensure access to medicines, especially ARVs, stakeholders need to operate effectively in complex, interdependent networks of organizations and systems across the public, private and non-profit sectors [27,56,57].

### Indications of impact

There is no global health strategy or policy in South Africa which enumerates a set of actions against which indicators will be developed and progress measured. However, issues of global health are reflected in South Africa’s ‘National Development Plan: Vision 2030’ and the Department of Health’s ‘Strategic Plan: 2014–2019’, which have set out strategic objectives and sets of actions against which indicators will be assessed and progress measured [21,58]. Furthermore, South Africa’s global health priorities are reflected in the Oslo Declaration and various BRICS Ministerial Declarations on health. The review of the literature suggests that South Africa’s impact could be categorized into six areas, namely: normative change, informal norms and power, establishment of new institutions, health impact, market impact and financial impact.

This study suggests that the main reason for South Africa’s positive impact on global health has been its commitment to health as a human right, as enshrined in the South African Constitution, the Bill of Rights and the National Health Act of 2003 [21,23,31]. This positive impact has manifested as a number of necessary normative changes, having been shaped in both law (national and international) and international relations. Furthermore, South Africa’s tenets of human dignity, equity, the advancement of human rights and the promotion of individual freedoms have advanced the nation’s reputation as a respected global player [27,50].

With respect to informal norms and power, various South African political and technical experts have been invited to chair or participate in numerous technical and advisory committees established by multilateral organizations. As one of the state interviewees in this study explained:

WHO for example when they invite people from the [SA] Department to attend their meeting to give strategic direction, then you know it means they value your input because you have done a good work at home or that you have a lot of influence by the people … many of the reasons they ask me to chair for example, it’s is not because as an individual, because they know if South Africa then adopt this thing, the rest will follow … You will see now because DG has been chairing EB, you will see that South Africa’s esteem in the world have gone up.

With respect to the establishment of institutions, this study suggests that South Africa’s overseas development cooperation has recently been further strengthened through the establishment of the development-focused South African Development Partnership Agency (SADPA) and the Department of Health’s Development Cooperation Unit (DCU) [49,58,59]. This implies that South Africa’s global development engagements could be even further be strengthened by the establishment of a partnership between the DCU and SADPA. However, the SADPA’s activities in global health have not been prioritized or governed by any broad strategy. Several interviewees have highlighted the role played by South Africa in development cooperation:

The other way to measure the success of global health diplomacy is the extent to which people call South Africa to assist them, when there is a disaster so then you know that people think that … help them.

With respect to health impact, this article shows that the integration of health into South Africa’s foreign policy, along with the reform of various policy instruments, led to a decline in the number of AIDS deaths in South Africa from 320,000 in 2010 to 140,000 in 2014. Furthermore, the number of mother-to-child HIV transmissions dropped from 70,000 in 2004 to under 7000 in 2015 [60]. Thus, South Africa has been able to significantly improve the average life expectancy of its people in recent years [58].

With respect to market impact, this study shows that South Africa has successfully utilized certain TRIPS flexibilities, such as parallel importation, to bring about certain reforms (e.g. those to the Amendment Act of 1997) that have led to the provision of a sustainable supply of essential medicines [61,62]. South African has now established mechanisms by which to implement all of the provisions contained in the WTO’s TRIPS flexibilities for public health. As South Africa’s Minister of Trade and Industry, Dr Davies, emphasized during a WTO meeting in April 2016:

First, following many delays, we have recently ratified the WTO Paragraph 6 mechanism that allows the issuance of compulsory licenses for export of medicines to countries that lack pharmaceutical manufacturing capacity. [63]

Through its Competition Law, South African government has been able to increase its local generic competition when several of its pharmaceutical manufacturers entered into voluntary licensing arrangements [61]. For example, South African firm Aspen Pharmacare has participated in several joint ventures with multinationals to manufacture and distribute branded and generic pharmaceuticals for both South African and African markets [64].

With respect to financial impact, this study suggests that the threat of compulsory licensing aided South Africa in its price negotiations with pharmaceutical companies, and was also successfully harnessed to obtain significant discounts from a number of brand manufacturers [65]. Furthermore, by reforming its drug tendering systems, South Africa has saved millions of dollars that can be better spent on expanding the nation’s treatment programmes. South Africa’s overall ARV expenditure fell by 53% between 2011 and 2012, generating estimated savings of about $685 million [66,67].

## Discussion

This study has illustrated the social, political, economic and institutional preconditions (and the complex interplay among them) that are necessary for successful GHD processes. It has also shown the extent to which international responses and collaboration can contribute to the efficacy of these processes. For example, evidence presented in this article shows that South Africa’s domestic HIV/AIDS constituencies, in partnership with the global advocacy movement, were influential in mobilizing support for universal access to ART on a global scale [68,69].

It has been demonstrated that political actors such as civil society, transnational networks, the global private sector, philanthropic foundations, ‘think tanks’ and a much wider range of countries than ever before now possess the necessary means for constructive participation in global health governance [69,70]. This article illuminates the significant role of health partnerships in facilitating access to medicines (especially ARVs) in South Africa – a country with limited public resources and therefore a particularly acute interest in the potential health benefits of successful GHD processes.

This article has confirmed the findings of other studies which found that in South Africa, integration of health into foreign policy was informed by a series of ‘focused events’ (such as the 13th International AIDS Conference, the Global March for HIV/AIDS Treatment, the PMA case in 2001, the Hazel Tau case and the Oslo Declaration), which led to ‘a turning point in acceptance of the right of access to treatment for people in Africa and other developing countries’ and health as a foreign policy issue [69,70]. Problems that have had to be addressed include South Africa’s historical context of democratization, oppression and apartheid, acute political and social inequalities, and the socio-economic challenges of severe poverty and extreme inequality in living standards, income and opportunity. Based on these challenges, South Africa has worked towards a rule-based multilateral system that is fair, balanced and inclusive, and the establishment of a new discourse focused on the significance of health for development and economic growth [69]. Multilateralism can therefore be seen as a key ingredient of positive change, even if many multilateral focused events have not been taken far enough to be significantly influential. Our study further supports previous studies advocating that the PMA case of 2001 was instrumental in laying the foundations for the negotiations and ultimate adoption of the Doha Declaration of 2001 [69].

This study has also confirmed suggestions in the literature that the integration of health into South African foreign policy has been made possible through interactions between domestic and international state and non-state actors [70,71]. South Africa now has the means to participate in global health governance, and there is a clear shift of power and a trend towards flexible alliance building in this area, indicative of a departure from Western dominance [72,73]. The South African health activism community, in consultation with transnational activism networks and ‘significant others’ such as the Brazilian government (‘policy community’), has advocated for broader access to affordable ARVs and healthcare services. Illustrative of their growing power, these national and global AIDS movements have been able to provide high-quality expertise, conduct research, provide policy analyses and engage in advocacy, as was the case during the PMA case in South Africa during 2001 and the Khayelitsha pilot project [74]. This Khayelitsha pilot project has highlighted the importance of community mobilization and innovation in facilitating scale-up projects for HIV prevention in South Africa.

There is a substantial growth of discursive and resource-based power in South Africa, exemplified by the country’s involvement in alliances such as BRICS and IBSA. The IBSA Facility Fund and BRICS Bank are likely to play a major role in new models of global health governance and financing [75–77]. For example, the IBSA Facility Fund, with US $3 million targeted annually for South–South development, has been a key in driving the change of roles in South–South cooperation. Thus, as with many other traditional structures, the Organisation for Economic Co-operation and Development’s (OECD’s) Development Assistance Committee model of dividing the world into ‘donors’ and ‘recipients’ or ‘subjects’ and ‘objects’ of development is losing relevance. Once again, the need becomes apparent for a new and a more systemic way of understanding the interface of health, development and wealth on a global scale, including new structures and novel solutions to twenty-first-century challenges.

The HIV/AIDS epidemic was clearly a key turning point or ‘cosmopolitan moment’ in South Africa’s approach to health; this was the factor that led to concrete government action to address global health issues [69,74]. In particular, reducing the cost of ART was the most important motivation for active engagement in GHD. The South African government is justifiably worried about the 3.4 million South Africans who were on ART by end of 2015, and an added concern is the affordability of second line and third line ARVs as more people develop resistance to first line drugs [69,74]. Furthermore, South Africa is worried about keeping them on treatment and thus increasing the burden with respect to the provision of medicines to all people living with HIV.

The policy stream was achieved through the development of a series of breakthrough policy reforms such as provision of free ARV medicines to people eligible for treatment (based on the 2010 WHO ART guidelines), reformation of drug tendering systems and the launch of the National Health Insurance policy. The policy stream was established by ensuring that HIV/AIDS treatment is among the core issues to be addressed and furthered by the Zuma administration. The South African national ART programme has already been given partial credit for the significant increase in South Africa’s life expectancy.

While diplomacy has traditionally been seen as the preserve of the political elite, this study has demonstrated that South Africa’s non-state actors such as the health activist community, academia and the private sector have successfully managed to leverage their organizational and legal resources to achieve wider access to affordable ARVs and healthcare services in South Africa. More broadly, successful use of ‘transnational issue networks’ has been an important aspect of South African engagements in GHD [72,73]. Notwithstanding these observations, this study has confirmed the results of other research that GHD policy processes are still subject to traditional political power dynamics [78,79].

Our study illustrates that the cross-sectoral nature of health policy problems is developing according to two trends. The first trend, ‘transgovernmentalism’ [80], is the development of policies by international networks of bureaucrats, lawyers, agencies and so on. The changing roles of health and foreign ministries are accompanied by a loss of autonomy. The second trend follows the increased involvement of international, private and transnational actors in the formation of foreign policies that integrate public health. Health and foreign ministries not only are losing authority to other ministries, but also need to consider and cooperate with non-state actors [72,73]. This study further demonstrated a tendency towards ‘denationalization’. Transnational actors such as Doctors Without Borders, Oxfam and the Gates Foundation are a force to be reckoned with in international politics. Transnational actors are influential in global health debates, and in the case of South Africa, most notably in the case of the Khayelitsha project, they were involved in the development and implementation of health policies.

## Conclusion

In this paper, we use four consecutive steps to illustrate how public health is integrated into foreign policy in South Africa. First, South Africa is still focused inwards, and international engagement is driven by its domestic challenges (such as the burden of HIV/AIDS and TB). Secondly, owing to the transboundary nature of diseases, South Africa has begun to take an effective regional and global leadership role in GHD, especially in the areas of HIV/AIDS, TB and malaria control. Access to medicines and treatments for all individuals may be a potential entry point for South African engagement in GHD.

Thirdly, in the field of GHD, we show that transnational networks and/or actors may influence a government’s decision making by providing information and moving issues up the agenda. While transnational networks and/or actors have been influential in global health debates, GHD is still a state-centric process. Fourthly, we argue that GHD analyses need to incorporate non-state actors into their frameworks. Therefore, empirical research is needed that describes these changes and their impact on the integration of health into foreign policy.

## Paper context

Little empirical research has been conducted to inform arguments for the integration of global health into foreign policy by state and non-state actors. This study seeks to address this knowledge gap regarding developing an understanding of how global health is integrated into foreign policy in South Africa, using the lens of access to antiretroviral medicines. We demonstrate that South Africa has been able to move towards ‘transgovernmentalism’ and ‘denationalization’, whereby health ministries lose their authority to other sectors and non-state actors. We argue that global health diplomacy scholars may want to consider whether their analytical foci and bedrock assumptions should be amended to reflect twenty-first-century developments such as the transnationalization of health governance.
